# Changes in metabolic and hormonal profiles during transition period in dairy cattle – the role of spexin

**DOI:** 10.1186/s12917-021-03069-4

**Published:** 2021-11-20

**Authors:** Robert Mikuła, Ewa Pruszyńska-Oszmałek, Marcin Pszczola, Justyna Rząsińska, Maciej Sassek, Krzysztof W. Nowak, Leszek Nogowski, Paweł A. Kołodziejski

**Affiliations:** 1 Department of Animal Nutrition, Poznań University of Life Sciences, Wołyńska 33, 60-637 Poznań, Poland; 2 Department of Animal Physiology, Biochemistry and Biostructure, Poznań University of Life Sciences, Wołyńska 35, 60 –, 637 Poznań, Poland; 3 Department of Genetics and Animal Breeding, Poznań University of Life Sciences, Wołyńska 33, 60-637 Poznań, Poland

**Keywords:** Dairy cow, Transition period, Energy balance, Spexin

## Abstract

**Background:**

This study aimed to evaluate spexin as a novel blood marker and to describe the relationship of this peptide with selected biochemical metabolites measured during the transition period in dairy cows. Additionally, mRNA expression of the spexin gene as well as spexin receptors – galanin receptor type 2 and galanin receptor type 3, was investigated in several bovine tissues. Blood samples were collected at weekly intervals starting at 21 days before the estimated parturition day until 21 days in milk to determine concentrations of spexin, nonesterified fatty acids, β-hydroxybutyrate acid, total and active ghrelin, progesterone, glucose, insulin, IGF–I, triglycerides, cholesterol, leptin, corticosterone and 17-β-estradiol as well as the activity of aspartate transaminase, alkaline phosphatase and gamma-glutamyl transferase.

**Results:**

Spexin concentration decreased from 21 d before parturition to calving day and next it rose during the first 14 d of lactation. The lowest concentration of spexin was recorded on the calving day and it differed from the mean level of this peptide before parturition as well as postpartum. Moreover, differences were observed between mean spexin concentrations before and after calving. Spexin levels were moderately negatively correlated with NEFA (*r* = − 0.39) and total ghrelin contents (*r* = − 0.41), weakly correlated with BHBA (*r* = − 0.35) while they showed a moderate positive relationship with progesterone concentrations (*r* = 0.42). Moreover, we detected that mRNA expression of GALR2, GALR3 and SPX is present in various bovine tissues (kidney, bowel, rumen, spinal cord, lung, skeletal muscle, liver, heart, fat and spleen).

**Conclusion:**

A negative correlation between spexin concentration and NEFA, BHBA and total ghrelin contents as well as a positive relationship with levels of progesterone, metabolites and hormones, which are key players in the dairy cow transition period, may confirm an important function of this peptide in metabolism regulation. Thus measurement of spexin concentration could provide useful supplementary information for dairy cow herd health monitoring.

## Background

The transition period is a very important time in dairy cattle production and can affect the metabolic and health status of dairy cows as well as their reproductive and yield performance in the consecutive lactation [1]. A decrease in dry matter intake around the time of calving may lead to a negative energy balance in early lactation, which is probably one of the main problems with metabolic homeostasis in dairy cows. Excessive lipolysis and rising levels of nonesterified fatty acids (NEFA) in dairy cows’ blood during negative energy balance are often associated with the accumulation of triglycerides (TG) in hepatocytes and impairment of liver function resulting in an elevated ketone production [2]. Moreover, increased blood NEFA levels could have a negative effect on oocyte development and reproductive performance [3]. Sordillo and Raphael [4] stated that a progressive increase in the blood NEFA level before calving may be considered a significant factor affecting inflammatory responses of transition cows. Higher blood NEFA concentrations might be related to an increased risk of displaced abomasum [5], metritis and retained placenta [6], mastitis [7] and lower milk yield [8], while they may also result in early-lactation culling risk [9].

Interest in the different blood indices that may be used to describe various metabolic conditions and poor adaptation to negative energy balance (NEB) has greatly increased in the recent years [10–14]. Many authors defined reference or cut-off levels before parturition and during fresh lactation to estimate the risk of metabolic diseases; however, new potential negative energy balance indicators are being searched for [13, 15, 16]. This search for such a blood marker is necessary and justified, as it would contribute to increased sustainability of milk production.

**Fig. 1:**
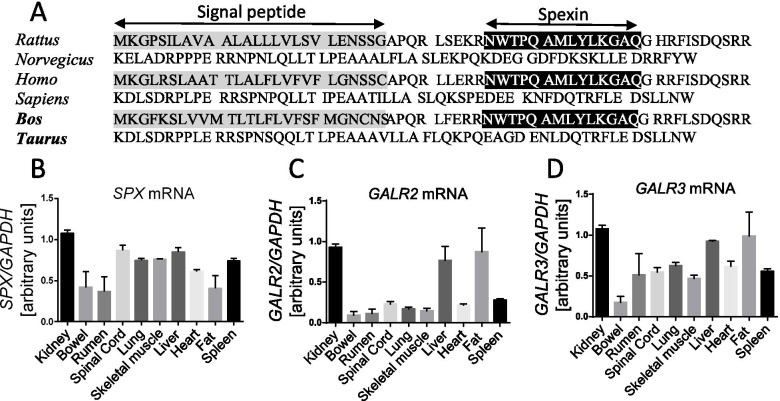
The sequence of SPX in animals and expression of SPX and its receptors in bovine tissues: comparison of SPX protein sequences reported in rat: human: and bovine (**A**), mRNA expression of SPX (**B**), GALR2 (**C**) and GALR3 (**D**) genes in different types of bovine tissues, kidney, bowel, rumen, spinal cord, lung, skeletal muscle, liver, heart, fat, spleen (n-5)

For this reason in the last few years several novel peptides, hormones or other biologically active substances have been suggested as potential substances involved in metabolism regulation, which may serve as indicators of the metabolic state in the future. One of them, spexin (SPX), is a very conservative peptide discovered in 2007 in an in-silico study by Mirabeu et al. [17]. Two years later the gene coding 116 aa spexin precursor containing 14 aa spexin was identified by the Somnez research group [18] (a comparison of the human, rat, and bovine SPX sequences is presented in Fig. 1 A). The biological activity of SPX is regulated via two subtypes of the galanin receptor: galanin receptor 2 (GALR2) and galanin receptor 3 (GALR3) [19]. Until now, most studies on the functions of spexin in metabolism control have been conducted on fish, rodents and humans. It is known that spexin is involved in the regulation of adipocyte metabolism, it inhibits food intake, insulin secretion and enhances bowel movement [20–22]. However, the role of SPX in bovine physiology is unknown.

The aim of this study was to evaluate spexin as a novel blood marker during the transition period, as well as describe variation and relationships of selected biochemical metabolites associated with negative energy balance. Moreover, it was decided in this study to investigate mRNA expression of the SPX gene as well as SPX receptors – GALR2 and GALR3 in different bovine tissues.

## Results

First we decided to investigate the expression of SPX and SPX receptors (GALR2 and GALR3) at the mRNA level in various bovine tissues. We found that the SPX (Fig. 1B) as well as GALR2 (Fig. 1C) and GALR3 genes (Fig. 1D) were expressed in all the investigated tissues (kidney, bowel, rumen, spinal cord, lung, skeletal muscle, liver, heart, fat and spleen). Due to the fact that this part of research was conducted on tissues from 5 animals, graphs only show the presence or absence of mRNA expression of the genes tested, which is the reason why it was decided not to determine statistical significance between tissues.

**Fig. 2:**
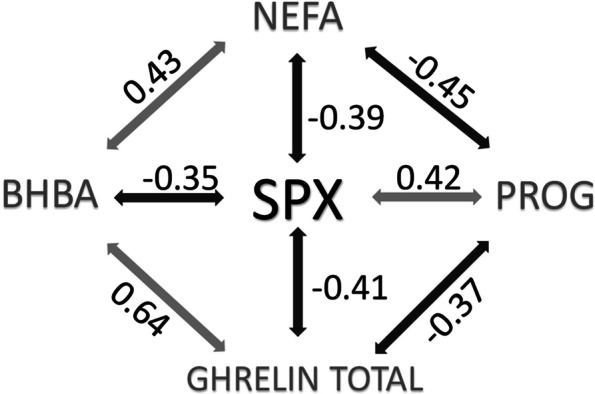
A model of relationships between spexin (SPX) and nonesterified fatty acids (NEFA), β-hydroxybutyrate acid (BHBA), ghrelin total and progesterone (PROG) (all correlations are significant)

**Fig. 3:**
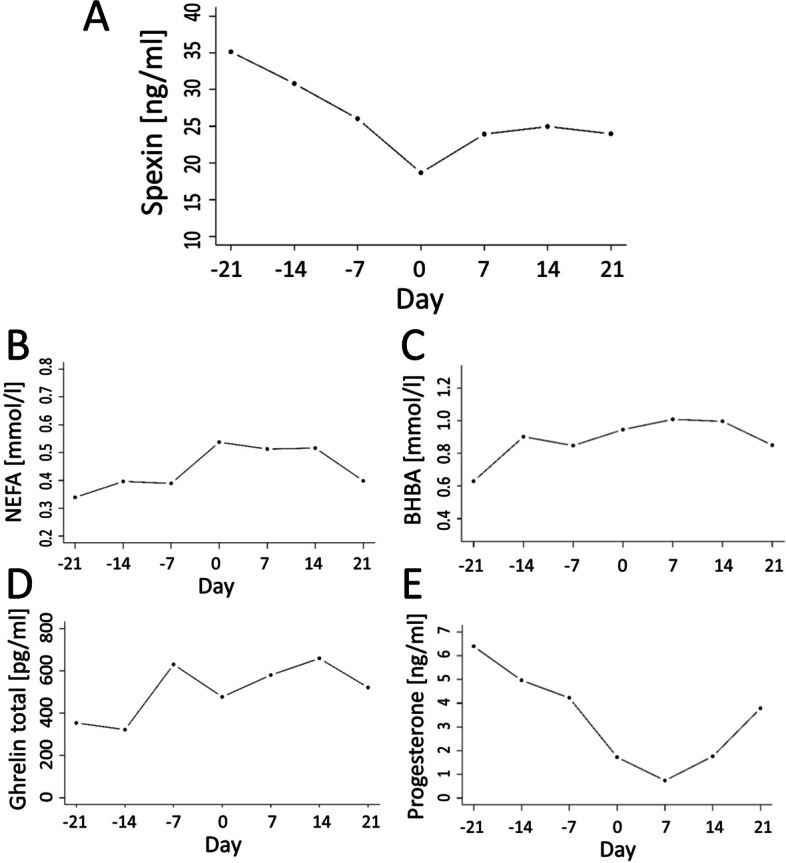
Changes in mean serum concentrations of spexin (A), NEFA (B), BHBA (C), ghrelin total (D) and progesterone (E) during transition period

Spexin concentration decreased from 21 d before parturition to calving day and next it increased during the first 14 d of lactation. The lowest concentration of spexin (18.45 ng/ml) was recorded on calving day (Fig. 3A) and it differed from the mean level of this peptide both before parturition (32.74 ng/ml; *P* < 0.01) and postpartum (24.46 ng/ml; *P* < 0.01). Moreover, differences were observed between mean spexin concentrations before and after calving (Table 1, *P* = 0.03). Spexin levels showed a moderate negative correlation with NEFA (*r* = − 0.39; *P* < 0.01), a weak correlation with BHBA (*r* = − 0.35; *P* < 0.01) and a moderate correlation with total ghrelin (*r* = − 0.41; *P* < 0.01), while they were positively moderately correlated with progesterone (*r* = 0.42; *P* < 0.01) (Fig. 2, Table 2).

**Table 1:** Means of blood biochemical metabolites during transition period in high yielding dairy cows (*n* = 10)

Blood indices	Time of sample collection			*Multiple comparison p-values*			*Overall P-value*
	pre	cal	post	pre-cal	cal-post	pre-post	
Spexin, ng/ml	32.74	18.45	24.46	< 0.001	0.025	< 0.001	< 0.001
NEFA, mmol/l	0.37	0.54	0.47	0.003	0.137	0.001	< 0.001
BHBA, mmol/l	0.78	0.94	0.96	0.049	0.954	0.001	0.001
Glucose, mg/dl	73.54	80.10	69.50	0.109	0.001	0.162	0.001
Insulin, ng/ml	0.71	0.81	0.42	0.888	0.037	0.008	0.002
IGF - I, ng/ml	118.64	128.45	135.64	NA	NA	NA	0.325
Triglycerides, mg/dl	38.62	38.84	34.68	0.994	0.052	0.032	0.008
Cholesterol, mg/dl	133.19	122.52	140.13	0.187	0.004	0.222	0.003
Ghrelin active, pg/ml	8.72	13.00	15.14	0.436	0.857	0.031	0.040
Ghrelin total, pg/ml	293.57	411.64	563.64	0.118	0.100	< 0.001	< 0.001
Leptin, ng/ml	19.25	17.44	18.73	NA	NA	NA	0.647
Corticosterone, ng/ml	1.17	8.51	7.68	0.019	0.935	< 0.001	< 0.001
Progesterone, ng/ml	5.59	1.73	2.37	NA	NA	NA	0.050
17 β-estradiol, pg/ml	106.61	117.11	19.88	0.990	0.144	0.006	0.003
AST, IU/l	61.15	55.22	62.41	NA	NA	NA	0.333
ALP, IU/l	35.68	38.06	33.76	NA	NA	NA	0.494
GGTP, IU/l	8.83	8.78	9.86	NA	NA	NA	0.440

*NA* multiple comparisons not performed

*NEFA* nonesterified fatty acid, *BHBA* β-hydroxybutyrate acid, *IGF – I* insulin-like growth factor I, *AST* aspartate transaminase: *ALP* alkaline phosphatase, *GGTP* gamma-glutamyl transferase, pre – mean concentration from -21d to -7d: cal – concentration at calving day, post – mean concetration from 7 to 21 day in milk

**Table 2:** Correlations of spexin and spexin related methabolites during transition period in high yielding dairy cows (*n* = 10)

Trait 1	Trait 2	Repeated measures correlation	*P-value*
Spexin, ng/ml	NEFA, mmol/l	−0.393	0.005
Spexin, ng/ml	BHBA, mmol/l	−0.346	0.012
Spexin, ng/ml	Ghrelin total, pg/ml	−0.405	0.005
Spexin, ng/ml	Progesterone, ng/ml	0.421	0.003
NEFA, mmol/l	BHBA, mmol/l	0.430	0.002
NEFA, mmol/l	Corticosterone, ng/ml	0.296	0.042
NEFA, mmol/l	IGF - I, ng/ml	0.495	< 0.001
NEFA, mmol/l	Ghrelin total, pg/ml	0.349	0.016
NEFA, mmol/l	Insulin, ng/ml	−0.537	0.000
NEFA, mmol/l	Progesterone, ng/ml	−0.446	0.002
NEFA, mmol/l	Leptin, ng/ml	−0.435	0.002
BHBA, mmol/l	IGF - I, ng/ml	0.368	0.009
BHBA, mmol/l	Ghrelin total, pg/ml	0.640	< 0.001
BHBA, mmol/l	Insulin, ng/ml	−0.452	0.002
BHBA, mmol/l	Glucose, mg/dl	−0.397	0.004
BHBA, mmol/l	Progesterone, ng/ml	−0.338	0.018
BHBA, mmol/l	TG, mg/dl	−0.288	0.050
Ghrelin total, pg/ml	Progesterone, ng/ml	−0.373	0.013
Progesterone, ng/ml	17 β -estradiol, pg/ml	0.363	0.023
Progesterone, ng/ml	Insulin, ng/ml	0.413	0.007
Progesterone, ng/ml	IGF - I, ng/ml	−0.453	0.001
Progesterone, ng/ml	Corticosterone, ng/ml	−0.609	< 0.001
Progesterone, ng/ml	ALP, IU/l	0.327	0.022

*NEFA* nonesterified fatty acid, *BHBA* β-hydroxybutyrate acid, *IGF – I* insulin-like growth factor I, *AST* aspartate transaminase, *ALP* alkaline phosphatase:

The NEFA level increased from 21 d before calving and it was higher to 14 days in milk (Fig. 3B). Mean prepartum NEFA concentration was lowest (0.37 mmol/l) and differences were recorded in comparison to that at calving day (0.54 mmol/ll) and the mean postpartum level (0.47 mmol/l; Table 1, *P* ≤ 0.05). NEFA levels were positively moderately correlated with BHBA (*r* = 0.43; *P* < 0.01), a weak correlation was found with corticosterone (*r* = 0.30; *P* < 0.05), a moderate correlation with IGF-I (*r* = 0.50; *P* < 0.01) and a weak correlation with total ghrelin (0.35; *P* < 0.05), whereas they were negatively moderately correlated with insulin (*r* = − 0.54; *P* < 0.01), progesterone (*r* = − 0.45; *P* < 0.01) and leptin (*r* = − 0.44; *P* < 0.01) (Fig. 2, Table 2).

BHBA concentration increased from 21 d before parturition to 7 days in milk (Fig. 3C), with the mean prepartum BHBA concentration being lowest (0.78 mmol/l) compared to calving day (0.94 mmol/l) and the mean postpartum level (0.96 mmol/l) (Table 1, P ≤ 0.05). BHBA concentration showed positive moderate correlations with IGF-I (*r* = 0.37; *P* < 0.01), total ghrelin (*r* = 0.64; *P* < 0.01) and negative moderate correlations with insulin (*r* = − 0.45; *P* < 0.01) and glucose (*r* = − 0.40; *P* < 0.01) as well as weak correlations with progesterone (*r* = − 0.34; *P* < 0.05) and triglycerides (*r* = − 0.29; *P* < 0.05) (Fig. 2, Table 2).

The total ghrelin concentration during the transition period was variable. A mild increase in total ghrelin concentration was observed during the first 14 d of the transition period, followed by a decrease up to calving day, as well as differences between pre- and post-parturition concentrations (293.6 pg/ml) vs. 563.6 pg/ml) of this hormone (Fig. 3D, Table 1). Total ghrelin concentration was only moderately correlated with spexin, NEFA and BHBA, as well as progesterone levels (*r* = 0.37; *P* < 0.01) (Table 2).

A decrease of progesterone concentration was observed from 21 d before parturition to 7 day in milk, followed by an increase (Fig. 3E), but differences between mean concentrations before (5.59 ng/ml) and after calving (2.37 ng/ml) were not confirmed statistically (Table 1, *P* > 0.05). Progesterone levels, apart from their correlations with spexin, NEFA, BHBA and total ghrelin concentrations, were positively moderately correlated with 17 β-estradiol (*r* = 0.36; *P* < 0.05), insulin (*r* = 0.41; *P* < 0.01), weakly correlated with ALP (*r* = 0.33; *P* < 0.05), while the relationship with IGF-I and corticosterone was moderately negative (*r* = − 0.45; *P* < 0.01, *r* = − 0.61; *P* < 0.01) (Tables 2 and 3).

**Table 3:** Correlations of other metabolites indirectly connected with spexin during transition period in high yielding dairy cows (*n* = 10)

Trait 1	Trait 2	Repeated measures correlation	*P-value*
Glucose, mg/dl	IGF - I, ng/ml	−0.312	0.029
Glucose, mg/dl	Insulin, ng/ml	0.547	< 0.001
Insulin, ng/ml	17 β -estradiol, pg/ml	0.660	< 0.001
IGF - I, ng/ml	Insulin, ng/ml	−0.536	< 0.001
TG, mg/dl	Glucose, mg/dl	0.424	0.003
TG, mg/dl	ALP, IU/l	0.288	0.047
TG, mg/dl	Ghrelin active, pg/ml	−0.422	0.005
TG, mg/dl	Insulin, ng/ml	0.394	0.013
TG, mg/dl	17 β -estradiol, pg/ml	0.563	< 0.001
Cholesterol, mg/dl	Ghrelin active, pg/ml	0.473	0.001
Leptin, ng/ml	Insulin, ng/ml	0.446	0.003
Leptin, ng/ml	17 β-estradiol, pg/ml	0.324	0.039
Corticosterone, ng/ml	IGF - I, ng/ml	0.373	0.009
Corticosterone, ng/ml	Ghrelin active, pg/ml	0.306	0.041
Corticosterone, ng/ml	Insulin, ng/ml	−0.308	0.045
Corticosterone, ng/ml	17 β -estradiol, pg/ml	−0.448	0.003
AST, IU/l	ALP, IU/l	0.422	0.002
AST, IU/l	IGF - I, ng/ml	0.339	0.020
GGTP, IU/l	IGF - I, ng/ml	−0.342	0.018
GGTP, IU/l	17 β -estradiol, pg/ml	−0.416	0.007

*NEFA* nonesterified fatty acid, *BHBA* β-hydroxybutyrate acid: *IGF* I - insulin-like growth factor I, *AST* aspartate transaminase, *ALP* alkaline phosphatase, *GGTP* gamma-glutamyl transferase

**Fig. 4:**
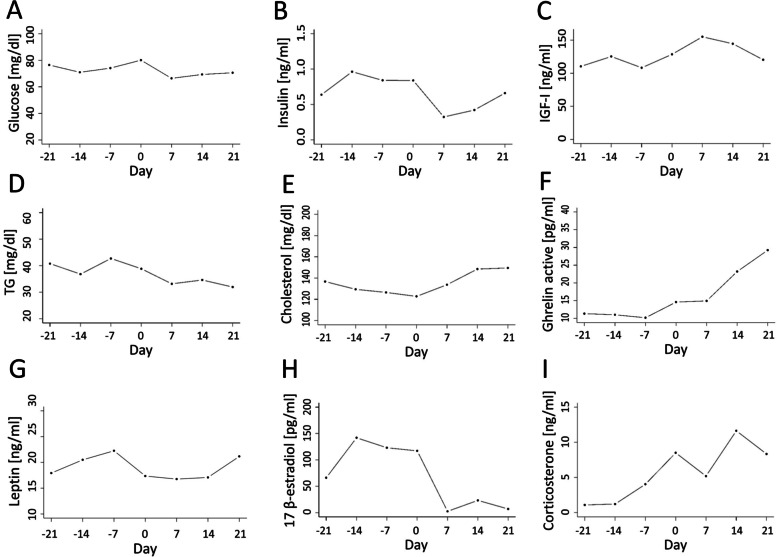
Changes in mean serum concentrations of glucose (**A**), insulin (**B**), IGF - I (**C**), triglycerides (**D**), cholesterol (**E**), ghrelin active (**F**), leptin (**G**), corticosterone (**H**) and 17 β-estradiol (I) during transition period

Blood glucose concentration reached the highest level on calving day (Fig. 4A); however, no differences were observed in comparison to mean glucose concentrations recorded pre- (73.54 mg/dl) and postpartum (69.50 mg/dl; Table 1, *P* > 0.05). Relationships between glucose levels and other blood indices are shown in Tables 2 and 3.

The lowest insulin concentration was recorded at 7 day in milk (Fig. 4B), while differences were confirmed statistically between the mean postpartum level of insulin (0.42/ng/ml) and the mean prepartum level (0.71 ng/ml) of these indices as well as those recorded on calving day (0.81 ng/ml) (Table 1, *P* ≤ 0.05). The correlations of insulin concentration with other blood markers are shown in Tables 2 and 3.

The highest peak of the IGF–I level was observed at 7 day in milk (Fig. 4C), whereas no differences were observed between these indices recorded on calving day (128.5 ng/ml) as well as pre- (118.6 ng/ml) or postpartum (135.6 ng/ml; Table 1, *P* > 0.05). The correlation of IGF-I concentration with other blood markers is shown in Tables 2 and 3.

The highest triglyceride concentration was recorded at 7 d before calving and next it decreased (Fig. 4D), which was confirmed by the mean prepartum triglyceride level (38.62 mg/dl), being higher compared to that postpartum (34.68 mg/dl; Table 1, P ≤ 0.05). The correlations of triglyceride levels with other blood markers are shown in Tables 2 and 3.

Cholesterol concentration decreased until calving day, followed by an increase in this blood marker (Fig. 4E). Differences were observed between cholesterol levels recorded at calving day (122.5 mg/dl) and from 7 to 21 days in milk (140.1 mg/dl; Table 1, P ≤ 0.05). Correlations of cholesterol concentration with other blood markers are shown in Table 3.

A mild decrease in the concentration of active ghrelin was observed during the first 2 weeks of the transition period, followed by an increase from 7 d before calving to 21 day in milk, with differences recorded between mean prepartum (8.72 pg/ml) and postpartum levels (15.14 pg/ml; Fig. 4F, Table 1, *P* = 0.03). Correlations of active ghrelin concentration with other blood markers are shown in Table 3.

Leptin concentration increased before parturition reaching the peak level at 7 d prepartum, followed by a decrease until 7 day in milk and a subsequent increase (Fig. 4G). There were no differences in leptin levels between the prepartum period and calving day, calving day and postpartum, prepartum and postpartum periods (19.25 ng/ml, 17.44 ng/ml, 18.73 ng/ml, respectively; Table 1, *P* > 0.05). Correlations between leptin concentration with other blood markers are shown in Tables 2 and 3.

An increase was observed in corticosterone concentration from 21 d before parturition to calving (Fig. 4I). The highest corticosterone concentration was recorded at 14 d in milk, with the mean postpartum level (7.68 ng/ml) differing from the prepartum values (1.17 ng/ml) of this hormone (Table 1, *P* ≤ 0.01). Correlations of corticosterone level with other blood markers are shown in Tables 2 and 3.

An increase was found in 17 β-estradiol levels from 14 d before calving to 7 day in milk (Fig. 4H), with differences observed between mean concentrations of 17 β-estradiol before (106.6 pg/ml) and after parturition (19.88 pg/ml) (Table 1, P ≤ 0.01). Correlations of 17 β-estradiol concentration with other blood markers are shown in Tables 2 and 3.

**Fig. 5:**
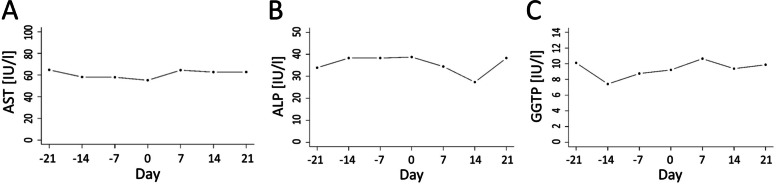
Changes in mean serum activity of AST (**A**), ALP (**B**), and GGTP (**C**) during transition period

There were no differences in the activities of AST, ALP and GGTP before and after calving (Fig. 5A - C, Table 1, P > 0.05). The correlations between activities of AST, ALP and GGTP and other blood markers are shown in Tables 2 and 3.

## Discussion

In our study we decided to examine the expression of SPX and its receptors at the mRNA level. Previous research conducted on other species such as rats, mice and humans showed systemic SPX expression [23, 24]. Our research also showed that SPX mRNA was detected in bovine kidney, intestinal, rumen, spinal cord, lung, skeletal muscle, liver, heart, fat and spleen tissues. In zebrafish and female goldfish, spexin mRNA was highly expressed in the brain and ovaries, which suggests that spexin plays a role in regulating the reproductive function [25, 26]. That is why we decided to investigate changes in SPX concentration during the transition period.

Research conducted on laboratory animals showed that spexin is a potent regulator of lipid metabolism [20, 22]. It is also known that a key area in the biology of transition cows is related to lipid metabolism [27] and strategies to reduce the extent of negative energy balance [28]. We hypothesised that spexin concentration may change during the transition period and that this peptide is correlated with selected blood markers used to describe the metabolic condition and adaptation to NEB in dairy cows. This study presents differences between mean spexin concentration before and after calving during the transition period. Spexin level decreased at the beginning of the transition period from 21 d before parturition to calving day, which was the lowest concentration point and next it rose during the first 14 d of lactation. Additionally, differences were observed in mean spexin concentration before and after calving, together with negative correlations between spexin and NEFA, BHBA and total ghrelin, as well as a positive relationship with progesterone, which suggests that this peptide is important during the transition of high-yielding cows from the dry period to lactation. The importance of spexin in transition cow metabolism is confirmed by the negative correlation between spexin and NEFA blood levels, which as is commonly known is elevated as a result of lipolysis and is strongly associated with a negative energy balance [2, 29]. The liver should oxidise NEFAs to produce energy or convert them back into triglycerides. However, during a negative energy balance NEFAs are often converted to ketone bodies, while as a result of excessive lipolysis in over-conditioned cows NEFAs are stored as fat droplets in hepatocytes. The two latter transformations are disadvantageous and lead to an increased risk of ketosis and fatty liver [30]. Thus the negative correlation between spexin and BHBA observed in the present study is probably indirect and reflects incomplete NEFA oxidation. Additionally, this hypothesis seems to be confirmed by a positive relationship between NEFA and BHBA concentrations, which was similar to the results described by Seifi et al. [12]. Moreover, the positive correlations between NEFA and total ghrelin, IGF–I, BHBA and corticosterone as well as the negative relationships with insulin, progesterone and leptin levels highlight spexin as a peptide, which may play an important role in the regulation of metabolism in dairy cows.

Moreover, both NEFAs and BHBA, which are blood markers connected with NEB, are negatively correlated with spexin and total ghrelin levels. Ghrelin is a gastrointestinal peptide hormone involved in the regulation of feed intake. According to Bradford and Allen [31] it may be a negative energy balance indicator, which is consistent with the observation of Takahashi et al. [32], who argued that ghrelin causes increased insulin secretion. In blood, ghrelin is mostly present in a non-posttranslationally modified form (total), while a minor portion of ghrelin is acylated at Ser3 (active) [33]. We observed an increased concentration of ghrelin (both total and active) in the postpartum period. These changes may be the result of an increase in demand for feed during lactation, as it is known that ghrelin is the strongest peripheral stimulator of food intake in mammals [34]. In turn, Börner et al. [33] showed that plasma ghrelin level is positively associated with body fat, liver fat and milk fat contents, but not with feed intake in dairy cows after parturition. Compared to the increasing concentration of ghrelin after calving we noted a decreased level of SPX, which was confirmed by a negative correlation between both blood markers. Based on these results and research on other mammalian species it may be assumed that SPX has an opposite role in the regulation of food intake [22, 25]. Our previous research conducted on healthy and obese women also confirmed that the active ghrelin level is negatively correlated with SPX level [20]. These literature data, as well as the results obtained during this study provide grounds for the conclusion that there is an interaction between these hormones in the metabolism of mammals. In addition, as described by Börner et al. [33], recent literature sources present a growing body of evidence that ghrelin is more involved in lipid mobilisation and fat distribution between tissues, which is the key area in the biology of transition cows. Despite the negative relationship between spexin and total ghrelin, no correlation was confirmed between spexin and active ghrelin levels. This may probably result from the lack of changes in the activity of ghrelin-O-acyltransferase (GOAT) – an enzyme capable of acylating ghrelin in vivo [35]*.* However, this finding requires further analysis, in particular because such relationships have been previously described in rats [36].

Other investigated hormones involved in the regulation of feed intake through their effect on carbohydrate and lipid metabolism included insulin and IGF-I. Although no statistically significant changes were observed between the period before and after calving, a small increase was observed in IGF-I concentration at day 7 postpartum, while at the same time insulin levels were falling. These observations confirm previous studies [37, 38]. A low insulin level after parturition may be caused by an increased growth hormone (GH) secretion to preserve the metabolic homeostasis of energy-deficient dairy cows in early lactation. However, as previously mentioned, no changes in IGF-I concentration were observed, which seems surprising, because increased GH levels reduce liver secretion of IGF-I [39]. On the other hand, some research reported a lack of reduction in IGF-I level after calving in high-producing dairy cows [40]. It is also known that postpartum dairy cows enter a period of negative energy balance (NEB) associated with low levels of circulating IGF–I; however, as shown by Wathes et al. [41], these changes may be countered by proper nutrition and milking intensity. The level of glucose, positively correlated with insulin concentration, decreased during the first week after calving, followed by an increase starting from week 2. This finding is similar to an observation of Vazquez-Añon et al. [42], who explained that it may reflect the recovery of feed intake and the improving energy status of the cow. In turn, no differences in glucose concentration analysed pre- and postpartum, as well as its correlations only with BHBA, IGF–I, insulin and TG levels might confirm the thesis that glucose concentration is not an effective marker of energy balance. A similar point of view was shown by Seifi et al. [12], who claimed that although glucose is relevant for organ function, fetal growth and milk production, its level is an insensitive measure of energy status because it is subject to tight homeostatic regulation.

The concentration of triglycerides was decreased during the transition period, which was confirmed by the mean postpartum triglyceride level being lower than in the prepartum period. A similar observation indicating higher triglyceride concentrations during the dry period than post parturition was made by Seifi et al. [12]. The triglyceride uptake by the mammary gland for milk fat synthesis during lactation may affect an increase in this blood marker level. Additionally, according to Djoković et al. [43], increased lipogenesis and ketogenesis in the liver results in decreased triglyceride concentrations in the blood.

The decrease of cholesterol concentration up to calving day, as reported in the present study, is probably connected with the higher requirement of fetal tissues and maternal glands for steroid hormone synthesis. Kessler et al. [44] claimed that at the onset of lactation the levels of enzymes essential for cholesterol biosynthesis are increased to meet the increased requirement of this marker to export liver triglycerides in lactating dairy cows. However, the changes in the hepatic gene expression are not reflected in a rise of blood cholesterol concentration. A moderate increase of cholesterol concentration during the first 21 d of lactation, as observed in the present study, may confirm this thesis. Additionally, the physiological liver function may be estimated based on the lack of differences in the mean activities of AST, ALP, and GGTP before and after calving.

It is commonly known that progesterone is a key factor in the establishment and maintenance of reproduction. A decrease of the progesterone level before calving was observed and it is relevant for uterine contractions, it contributes to the onset of lactation and allows mammary epithelial tissue to respond to the lactogen complex [10]. A rise of progesterone concentration was observed during the first 21 d of lactation, which was similar to findings presented by Adriaens et al. [45] and Blavy et al. [46], who described milk progesterone curves during lactation. Progesterone plays a critical function after calving in supporting uterine function and is important for embryo development as well as early embryo loss [29, 47]. Therefore a positive progesterone correlation with spexin level underscores the role of spexin in dairy cow metabolism during the transition and fresh lactation periods.

From an endocrinological point of view, the limitation of this study is that it showed only a change in the SPX concentration during the transitional period in dairy cattle, rather than the direct effect of SPX. However, this is the first study regarding the spexin peptide in cows and our team intends to carry out further experiments to determine its influence on the organism of these animals as well as their dry matter intake and milk performance.

## Conclusion

The present study described novel information concerning a negative correlation between spexin and NEFA, BHBA and total ghrelin levels as well as a positive relationship with progesterone, metabolites and these hormones, which are key players in the metabolism of dairy cows in the transition period.

Spexin was shown to be an important factor in cattle metabolism during the close–up and early lactation periods. Thus measurement of spexin concentration could provide useful supplementary information for health monitoring in dairy cow herds.

Future studies should investigate the specific relationship between spexin level and dry matter intake, milk performance, fertility parameters in dairy cows, as well as electrolyte levels associated with body fluid distribution [48].

## Methods

### Animal management, experimental design and diets

All animal activites were conducted following the guidelines of the Polish Council for Animal Care (Act on the Protection of Animals Used for Scientific or Educational Purposes in Poland adopted on 15th January 2015 and according to earlier regulations) and are conventional practices for animal health assessment and monitoring; in particular, blood samples were collected during standard veterinary activites, while tissue samples were obtained from a slaughterhouse.

The experiment was performed at a commercial farm in Wielkopolska (Poland). Ten Polish Holstein multiparous high–yielding dairy cows were selected for the study, which covers from 21 days before parturition to 21 days of lactation (animals for blood serum analysis). Moreover, 5 different animals were used to investigate gene expression (SPX mRNA expression in bovine tissues). The nutritional values of the feed components were calculated based on the wet chemistry laboratory analyses of nutrient contents using the PrevAlim 3.23 software (Educagri/INRA, Theix, France). Dry matter was analysed in a binder dryer according to the AOAC 934.01 method [49]. Crude protein (CP) was measured by the Kjeldahl method (AOAC 976.06) in a Kjelfoss Automatic 16,210 apparatus [49]. Crude fibre (CF, PN-EN ISO 6865) [50], neutral detergent fibre (NDF, PN-EN ISO 16472:2007) [51] and acid detergent fibre (ADF, PN-EN ISO 13906:2009) [52] were determined using the Tecator Foss Fibertec System M. Crude ash (Ash) was collected after a sample was burnt in a Nobertherm oven (550 °C) (AOAC 942.05) [49]. The feeding diets were balanced according to the recommendations provided by the French National Institute for Agricultural Research and using the INRAtion 3.3 software (Educagri/INRA, Theix, France). Cows were fed a total mixed ration (TMR), which was served to the animals twice a day: at 7.00 AM and 3.00 PM. The animals from − 56 d to − 21 d before parturition were fed a far-off diet, which contained 31.7% straw, 22.6% maize silage, 20.8% alfalfa silage, 18.1% grass silage, 4% soybean meal, 1.6% rapeseed meal and 1.2% minerals and vitamins (0.70 feed unit for lactation (UFL)/kg dry matter (DM), 69 g protein digested in the small intestine (PDI)/kg DM, 54% neutral detergent fibre (NDF). The close-up diet covered the last 21 d before calving and contained 31.6% maize silage, 10% grass silage, 10% alfalfa silage, 9.2% brewer’s grain silage, 6.6% soybean meal, 6.6% triticale grain, 6.5% barley grain, 6.1% sugar beet pulp silage, 4.6% straw, 4.3% maize grain silage, 2.2% rapeseed meal and 2.3% minerals, vitamins and additives (0.87 UFL/kg DM, 101 g PDI/kg DM, 33% NDF). The fresh lactation diet was fed after calving and contained 25.8% maize silage, 11.4% alfalfa silage, 11% grass silage, 9.5% maize grain silage, 8% barley grain, 8% triticale grain, 6.8% brewer’s grain silage, 6.4% soybean meal, 4.5% sugar beet pulp silage, 3.3% rapeseed meal, 3.3% straw and 2% minerals and vitamins and additives (0.92 UFL/kg DM, 110 g PDI/kg DM, 28.7% NDF). All the diets ingredients are given in terms of dry matter. During the experiment the silages were analysed and verified once a week using the NIRS method (Foss InfraXact, Hilleroed, Denmark). Weekly forage and TMR representative samples were collected, frozen and stored (− 20 °C) for further pooled monthly analysis using wet chemistry methods. Based on crude protein, neutral detergent fibre and acid detergent fibre, crude ash of the feeds as well as the TMR were verified and recalculated monthly.

### Blood and tissue sample collection

Blood samples were obtained 3 h after morning feeding [53] at weekly intervals on days 21, 14, 7 before the estimated parturition day, on calving day and on days 7, 14, 21 post parturition. Calving time was estimated based on the 280-day pregnancy period and was predicted with approximately − 2/+ 1 day to real parturition. Blood was collected during a standard veterinary procedure from the jugular vein into tubes with polystyrene separating granules covered with a clot activator and then the aliquots were rotated in a centrifuge (3500 x g for 15 min at 4 °C) within 1 h. Next, serum was divided into small samples, frozen in 1.5 ml tubes and stored at − 20 °C for later biochemical and hormonal analyses [54, 55].

For SPX mRNA expression analyses samples of animal tissues were taken post-mortem in a slaughterhouse. Samples were collected from the animal within 30 min after sacrifice and frozen in liquid nitrogen.

### RNA isolation and real time PCR

Total RNA from tissues was isolated using the Tripure reagent according to the manufacturer’s recommendations as we previously described [20]. cDNA was synthesised using 1 μg of total RNA and the cDNA reverse transcription kit (Applied Biosystems, USA). The real time PCR reaction was run in 5× HOT FIREPol® EvaGreen qPCR Mix Plus (ROX) on the QuantStudio 12 K Flex™ Realtime PCR system (Life Technologies, Grand Island, NY, USA) using specific primers: spexin forward 5’ctgtctagaactttaggctttatttgc3’, spexin reverse 5’acactggagtgggttgctgt3’ (product size 61 bp); GALR2 forward 5’ctcgctcctccccttgat3’, GALR2 reverse 5’ctgcgagcggagtgatct3’ (product size 67 bp); GALR3 forward 5’cagcttcagcctggtcagtt3’ GALR3 reverse 5’acagacctcaaccctgatgg3’ (product size 60 bp); and glyceraldehyde 3-phosphate dehydrogenase gene (GAPDH) forward 5’ggcgtgaaccacgagaagtataa3’, GAPDH reverse 5′ ccctccacgatgccaaagt3’ (product size 119 bp) as a reference gene. The real time PCR program included 15 min of initial activation and a three-step amplification program (40 cycles): denaturation at 95 °C for 15 s, annealing at 62.0 °C for 37 s and elongation at 72 °C for 20 s. The specificity of reaction products was tested by determining the melting points (0.1 C/s transition rate). Relative gene expression was calculated by Delta Delta CT (ΔΔCT).

### Hormonal and metabolic profiles

Serum spexin was analysed using the Spexin / Neuropeptide Q (NPQ) (Human, Rat, Mouse, Bovine) RIA Kit according to the manufacturer’s instructions (Phoenix Pharmaceuticals, USA).

Serum cholesterol, triglyceride, glucose and β-hydroxybutyrate acid (BHBA) levels were determined using colorimetric assay kits (Pointe Scientific, USA) [56]. The levels of nonesterified fatty acids were calculated using the Wako kit (Wako Chemicals, USA). The concentrations of total and active ghrelin, progesterone, insulin, insulin-like growth factor I (IGF–I), leptin, corticosterone and 17-β-estradiol were analysed by means of a radioimmunoassay (RIA). Enzyme activity (aspartate aminotransferase – AST, alkaline phosphatase – ALP, and gamma-glutamyl transferase – GGTP) was determined using the kinetic method with the Pointe Scientific kits. Concentrations of hormones in blood serum were measured using an immunoassay (ELISA) or radioimmunoassay kits (RIA). Detailed descriptions of ELISA/RIA kits are given in Table 4. Optical density of samples (in the colorimetric assay and ELISA kits) was measured using a Synergy 2 microplate reader (Biotek, USA). Specific activity of the samples from the RIA method was measured using a 2470 Wizard2™ γ-counter (Perkin Elmer, USA).

**Table 4:** The list of assays used to determine metabolic and hormonal profiles

Target	Kit name	Sensitivity	Cat. No.	Manufacturer
Spexin	Spexin / NPQ (Human: Mouse: Bovine) - EIA Kit	0–100 ng/ml	EK-023-81	Phoenix Pharmaceuticals: USA
NEFA	NEFA-HR	0.01–4.0 mmol/l	434–91,795 434–91,995	Wako Chemicals: Germany
BHBA	β-Hydroxybutyrate	0.1–4.5 mmol/l	H 7587	Pointe Scientific: USA
Ghrelin (total)	Ghrelin (TOTAL) RIA	100–10,000 pg/mL	GHRA-88HK	Merck Millipore: USA
Progesterone	Progesterone Elisa kit	0-40 ng/ml	DNOV006	NovaTec: Germany
Glucose	Glucose Oxy	10–500 mg/dl	G7519	Pointe Scientific: USA
Insulin	Insulin-Specific RIA	2–200 μU/mL	HI-14 K	Merck Millipore: USA
IGF-I	IGF-1600 ELISA	0–600 ng/mL	EIA-4140	DRG: Germany
Triglycerides	Triglycerides DST	0–1000 mg/dl	T7531	Pointe Scientific: USA
Cholesterol	Cholesterol kit	0–700 mg/dl	C7510	Pointe Scientific: USA
Ghrelin (active)	Ghrelin (ACTIVE) RIA	10–2000 pg/mL	GHRA-88HK	Merck Millipore: USA
Leptin	Multi-Species Leptin RIA	1–50 ng/mL	XL-85 K	Merck Millipore: USA
Corticosterone	Corticosterone Double Antibody RIA Kit	0–1000 ng/ml	SKU 07120103	MP Biomedicals: USA
17β-estradiol	17 beta-Estradiol Elisa kit	0–600 pg/ml	DNOV003	NovaTec: Germany
AST	AST (SGOT) Liquid Reagents	0–500 IU/l	A7560	Pointe Scientific: USA
ALP	Alkaline Phosphatase	0–800 IU/l	A7505	Pointe Scientific: USA
GGTP	Gamma Glutamyl Transferase Reagent Set	0–1000 IU/l	G7571	Pointe Scientific: USA

*NEFA* nonesterified fatty acid: *BHBA* β-hydroxybutyrate acid: *IGF – I* insulin-like growth factor I: *AST* aspartate transaminase: *ALP* alkaline phosphatase: *GGTP* gamma-glutamyl transferase

### Statistical analyses

The significance of the differences between the precalving, calving and post-partum periods for most of the parameters was assessed using the mixed models with the lme4 package [57] available in the R software [58]. The model included the fixed effect of the period and a random effect of the animal and was solved using the maximum likelihood method. Three parameters, i.e. ghrelin active, total ghrelin and insulin, were normalised by log transformation before the analyses. Whenever, the variables had non-normal distribution and when the transformation to normal distribution was not possible, we used Generalized Linear Models. This was the case for progesterone and 17 β-estradiol. These variables were analyzed using generalised linear models with Gamma distribution and the log-link function available in the lme4 package [57].

The significance of the difference in the contrasts between the periods was obtained with the use of the lsmeans package [59] with the degrees of freedom approximated using the Kenward-Roger method [60], while the *p*-values were adjusted for multiple comparisons using the Tukey method. The means reported in the tables are backtransformed to an original scale of the measurements. The correlations between the parameters were calculated as repeated measures of correlations using the rmcorr package [61] available in the R software R Core Team [58] according to Bland and Altman [62]. The correlations were interpreted as: very strong correlation (±0.91 to ±1.00); strong correlation (±0.68 to ±0.90); moderate correlation (±0.36 to ±0.67); weak correlation (±0.21 to ±0.35); and negligible correlation (0 to ±0.20) [63, 64]. Correlations were considered significant when *p* ≤ 0.05.

## Data Availability

The datasets used and analysed during the current study are available from the corresponding author on reasonable request.

## Abbreviations

ADF: Acid detergent fibre
ALP: Alkaline phosphatase
AST: Aspartate aminotransferase
BHBA: β-hydroxybutyrate acid
CF: Crude fibre
CP: Crude protein
DM: Dry matter
GALR2: Galanin receptor 2
GALR3: Galanin receptor 3
GGTP: Gamma-glutamyl transferase
GH: Growth hormone
GOAT: Ghrelin-O-acyltransferase
IGF – I: Insulin-like growth factor I
NDF: Neutral detergent fibre
NEB: Negative energy balance
NEFA: Nonesterified fatty acids
PDI: Protein digested in the small intestine
SPX: Spexin
TG: Triglycerides
TMR: Total mixed ration
UFL: Feed unit for lactation

## References

[1] Roche JR, Meier S, Heiser A, Mitchell MD, Walker CG, Crookenden MA, et al. Effects of precalving body condition score and prepartum feeding level on production, reproduction, and health parameters in pasture-based transition dairy cows. J Dairy Sci. 2015;1–19. 10.3168/jds.2014-9269. 10.3168/jds.2014-9269 26233449

[2] Esposito G, Irons PC, Webb EC, Chapwanya A (2014). Interactions between negative energy balance, metabolic diseases, uterine health and immune response in transition dairy cows. Anim Reprod Sci.

[3] Walsh RB, Walton JS, Kelton DF, LeBlanc SJ, Leslie KE, Duffield TF (2007). The effect of subclinical ketosis in early lactation on reproductive performance of postpartum dairy cows. J Dairy Sci.

[4] Sordillo LM, Raphael W (2013). Significance of metabolic stress, lipid mobilization, and inflammation on transition cow disorders. Vet Clin North Am Food Anim Pract.

[5] Chapinal N, Carson M, Duffield TF, Capel M, Godden S, Overton M (2011). The association of serum metabolites with clinical disease during the transition period. J Dairy Sci.

[6] Ospina PA, Nydam DV, Stokol T, Overton TR (2010). Evaluation of nonesterified fatty acids and beta-hydroxybutyrate in transition dairy cattle in the northeastern United States: critical thresholds for prediction of clinical diseases. J Dairy Sci.

[7] Suriyasathaporn WS, Heuer CH, Noordhuizen-Stassen EN, Schukken YHS (2000). Hyperketonemia and the impairment of udder defense: a review. Vet Res.

[8] Ospina PA, Nydam DV, Stokol T, Overton TR (2010). Associations of elevated nonesterified fatty acids and β-hydroxybutyrate concentrations with early lactation reproductive performance and milk production in transition dairy cattle in the northeastern United States. J Dairy Sci.

[9] Roberts T, Chapinal N, Leblanc SJ, Kelton DF, Dubuc J, Duffield TF (2012). Metabolic parameters in transition cows as indicators for early-lactation culling risk. J Dairy Sci.

[10] Kurpińska A, Skrzypczak W (2020). Hormonal changes in dairy cows during periparturient period. Acta Sci Pol Zootech.

[11] Šamanc H, Gvozdić D, Fratrić N, Kirovski D, Djoković R, Sladojević Ž (2015). Body condition score loss, hepatic lipidosis and selected blood metabolites in Holstein cows during transition period. Anim Sci Pap Rep.

[12] Seifi HA, Gorji-Dooz M, Mohri M, Dalir-Naghadeh B, Farzaneh N (2007). Variations of energy-related biochemical metabolites during transition period in dairy cows. Comp Clin Path.

[13] Seifi HA, LeBlanc SJ, Leslie KE, Duffield TF (2011). Metabolic predictors of post-partum disease and culling risk in dairy cattle. Vet J.

[14] Shin E-K, Jeong J-K, Choi I-S, Kang H-G, Hur T-Y, Jung Y-H, et al. Relationships among ketosis, serum metabolites, body condition, and reproductive outcomes in dairy cows. Theriogenology. 2015;1–9. 10.1016/j.theriogenology.2015.03.014. 10.1016/j.theriogenology.2015.03.014 25872806

[15] Asl AN, Nazifi S, Ghasrodashti AR, Olyaee A (2011). Prevalence of subclinical ketosis in dairy cattle in the southwestern Iran and detection of cutoff point for NEFA and glucose concentrations for diagnosis of subclinical ketosis. Prev Vet Med.

[16] LeBlanc SJ, Leslie KE, Duffield TF (2005). Metabolic predictors of displaced abomasum in dairy cattle. J Dairy Sci.

[17] Mirabeau O, Perlas E, Severini C, Audero E, Gascuel O, Possenti R (2007). Identification of novel peptide hormones in the human proteome by hidden Markov model screening. Genome Res.

[18] Sonmez K, Zaveri NT, Kerman IA, Burke S, Neal CR, Xie X, et al. Evolutionary sequence modeling for discovery of peptide hormones. PLoS Comput Biol. 2009;5:e1000258 10.1371/journal.pcbi.1000258 PMC2603333 19132080

[19] Kim DK, Yun S, Son GH, Hwang JI, Park CR, Il KJ (2014). Coevolution of the spexin/galanin/kisspeptin family: Spexin activates galanin receptor type II and III. Endocrinology..

[20] Kolodziejski PA, Pruszynska-Oszmalek E, Micker M, Skrzypski M, Wojciechowicz T, Szwarckopf P (2018). Spexin: a novel regulator of adipogenesis and fat tissue metabolism. Biochim Biophys Acta - Mol Cell Biol Lipids.

[21] Lin CY, Zhang M, Huang T, Yang LL, Fu HB, Zhao L (2015). Spexin enhances bowel movement through activating L-type voltage-dependent calcium channel via galanin receptor 2 in mice. Sci Rep.

[22] Walewski JL, Ge F, Lobdell H, Levin N, Schwartz GJ, Vasselli JR (2014). Spexin is a novel human peptide that reduces adipocyte uptake of long chain fatty acids and causes weight loss in rodents with diet-induced obesity. Obesity..

[23] Porzionato A, Rucinski M, Macchi V, Stecco C, Malendowicz LK, De Caro R (2010). Spexin expression in normal rat tissues. J Histochem Cytochem.

[24] Gu L, Ma Y, Gu M, Zhang Y, Yan S, Li N (2015). Spexin peptide is expressed in human endocrine and epithelial tissues and reduced after glucose load in type 2 diabetes. Peptides..

[25] Zheng B, Li S, Liu Y, Li Y, Chen H, Tang H (2017). Spexin suppress food intake in zebrafish: evidence from gene knockout study. Sci Rep.

[26] Liu Y, Li S, Qi X, Zhou W, Liu X, Lin H (2013). A novel neuropeptide in suppressing luteinizing hormone release in goldfish, *Carassius auratus*. Mol Cell Endocrinol.

[27] Drackley JK (2010). Biology of dairy cows during the transition period: the final frontier?. J Dairy Sci.

[28] Cermakova J, Kudrna V, Simeckova M, Vyborna A, Dolezal P, Illek J (2014). Comparison of shortened and conventional dry period management strategies. J Dairy Sci.

[29] Garnsworthy PC, Lock A, Mann GE, Sinclair KD, Webb R (2008). Nutrition, metabolism, and fertility in dairy cows: 1. Dietary energy source and ovarian function. J Dairy Sci.

[30] Sordillo LM, Mavangira V (2014). The nexus between nutrient metabolism, oxidative stress and inflammation in transition cows. Anim Prod Sci.

[31] Bradford BJ, Allen MS (2008). Negative energy balance increases periprandial ghrelin and growth hormone concentrations in lactating dairy cows. Domest Anim Endocrinol.

[32] Takahashi H, Kurose Y, Kobayashi S, Sugino T, Kojima M, Kangawa K (2006). Ghrelin enhances glucose-induced insulin secretion in scheduled meal-fed sheep. J Endocrinol.

[33] Börner S, Derno M, Hacke S, Kautzsch U, Schäff C, Thanthan S (2013). Plasma ghrelin is positively associated with body fat, liver fat and milk fat content but not with feed intake of dairy cows after parturition. J Endocrinol.

[34] Delporte C (2013). Structure and physiological actions of ghrelin. Scientifica (Cairo).

[35] Sato T, Ida T, Nakamura Y, Shiimura Y, Kangawa K, Kojima M (2014). Physiological roles of ghrelin on obesity. Obes Res Clin Pract.

[36] Nakahara K, Nakagawa M, Baba Y, Sato M, Toshinai K, Date Y (2006). Maternal ghrelin plays an important role in rat fetal development during pregnancy. Endocrinology..

[37] Rhoads RP, Kim JW, Leury BJ, Baumgard LH, Segoale N, Frank SJ (2004). Insulin increases the abundance of the growth hormone receptor in liver and adipose tissue of periparturient dairy cows. J Nutr.

[38] Falkenberg U, Haertel J, Rotter K, Iwersen M, Arndt G, Heuwieser W (2008). Relationships between the concentration of insulin-like growth factor-1 in serum in dairy cows in early lactation and reproductive performance and milk yield. J Dairy Sci.

[39] Csillik Z, Faigl V, Keresztes M, Galamb E, Hammon HM, Tröscher A (2017). Effect of pre- and postpartum supplementation with lipid-encapsulated conjugated linoleic acid on reproductive performance and the growth hormone–insulin-like growth factor-I axis in multiparous high-producing dairy cows. J Dairy Sci.

[40] Tamadon A, Kafi M, Saeb M, Mirzaei A, Saeb S (2011). Relationships between insulin-like growth factor-I, milk yield, body condition score, and postpartum luteal activity in high-producing dairy cows. Trop Anim Health Prod.

[41] Wathes DC, Cheng Z, Fenwick MA, Fitzpatrick R, Patton J (2011). Influence of energy balance on the somatotrophic axis and matrix metalloproteinase expression in the endometrium of the postpartum dairy cow. Reproduction..

[42] Vazquez-Añon M, Bertics S, Luck M, Grummer RR, Pinheiro J (1994). Peripartum liver triglyceride and plasma metabolites in dairy cows. J Dairy Sci.

[43] Djoković R, Šamanc H, Jovanović M, Nikolić Z (2007). Blood concentrations of thyroid hormones and lipids and content of lipids in the liver in dairy cows in transitional period. Acta Vet Brno..

[44] Kessler EC, Gross JJ, Bruckmaier RM, Albrecht C (2014). Cholesterol metabolism, transport, and hepatic regulation in dairy cows during transition and early lactation. J Dairy Sci.

[45] Adriaens I, Saeys W, Huybrechts T, Lamberigts C, François L, Geerinckx K (2018). A novel system for on-farm fertility monitoring based on milk progesterone. J Dairy Sci.

[46] Blavy P, Friggens NC, Nielsen KR, Christensen JM, Derks M (2018). Estimating probability of insemination success using milk progesterone measurements. J Dairy Sci.

[47] Lonergan P (2011). Influence of progesterone on oocyte quality and embryo development in cows. Theriogenology..

[48] Cappai MG, Liesegang A, Dimauro C, Mossa F, Pinna W (2019). Circulating electrolytes in the bloodstream of transition Sarda goats make the difference in body fluid distribution between single vs. twin gestation. Res Vet Sci.

[49] AOAC International, 2005. Official methods of analysis of the AOAC. 18th Edition, Gaithersburg, MD, USA. 2005;:2005.

[50] Polish Norm PN-EN ISO 6865:2000. Animal feeding stuffs - Methods for analysis of animal feeding stuffs – Determination of crude fiber content – method with intermediate filtration. 2000;:6865.

[51] Polish Norm PN-EN ISO 16472:2007, Animal feeding stuffs - Determination of amylase- treated neutral detergent fibre content (aNDF). 2007;:16472.

[52] Polish Norm PN-EN ISO 13906:2008, Animal feeding stuffs - Determination of acid detergent fibre (ADF) and acid detergent lignin (ADL) contents. 2009;:13906.

[53] Mikuła R, Pruszyńska-Oszmałek E, Ignatowicz-Stefaniak M, Kołodziejski PA, Maćkowiak P, Nowak W (2020). The effect of propylene glycol delivery method on blood metabolites in dairy cows. Acta Vet Brno.

[54] Kolodziejski PA, Pruszynska-Oszmalek E, Sassek M, Kaczmarek P, Szczepankiewicz D, Billert M (2017). Changes in obestatin gene and GPR39 receptor expression in peripheral tissues of rat models of obesity, type 1 and type 2 diabetes. J Diabetes.

[55] Mikuła R, Pruszyńska-Oszmałek E, Kołodziejski PA, Nowak W (2020). Propylene glycol and maize grain supplementation improve fertility parameters in dairy cows. Animals..

[56] Mikuła R, Nowak W, Jaśkowski JM, Maćkowiak P, Oszmałek EP (2011). Effects of different starch sources on metabolic profile, production and fertility parameters in dairy cows. Pol J Vet Sci.

[57] Bates D, Mächler M, Bolker BM, Walker SC. Fitting linear mixed-effects models using lme4. J Stat Softw. 2015;67:1–48

[58] R Core Team. R Core Team 2014 R: A language and environment for statistical computing. R foundation for statistical computing. https://www.R-project.org/. 2015;:2015. http://www.r-project.org/.

[59] Lenth RV. Least-squares means: the R package lsmeans. J Stat Softw. 2016;69:1–33

[60] Kenward MG, Roger JH (1997). Small sample inference for fixed effects from restricted maximum likelihood. Biometrics.

[61] Bakdash JZ, Marusich LR (2017). Repeated measures correlation. Front Psychol.

[62] Bland j. M, Altman DG. (1995). Calculating correlation coefficients with repeated observations: part 2—correlation between subjects. BMJ..

[63] Raspa F, Tarantola M, Bergero D, Nery J, Visconti A, Mastrazzo CM (2020). Time-budget of horses reared for meat production: influence of stocking density on behavioural activities and subsequent welfare. Animals..

[64] Prion S, Haerling KA (2014). Making sense of methods and measurement: Pearson product-moment correlation coefficient. Clin Simul Nurs.

